# Supplementary material for: Genome Mining Reveals a Surprising Number of Sugar Reductases in Aspergillus niger
Source: J Fungi (Basel). 2023 Nov 24;9(12):1138. doi: 10.3390/jof9121138 (PMC10744612; doi:10.3390/jof9121138)
Supplement: Supplementary file 1 [file jof-09-01138-s001.zip › jof-2711422-supplementary-Figures S1-S6.pdf]

Genome mining reveals a surprising number of sugar reductases in *Aspergillus niger*

Astrid Müller [1], Li Xu [1], Claudia Heine [1], Tila Flach [1], Miia R. Mäkelä [2] and Ronald P. de Vries [1,*]

Supplemental Material.

Combines Supplemental Figures S1 – S6.

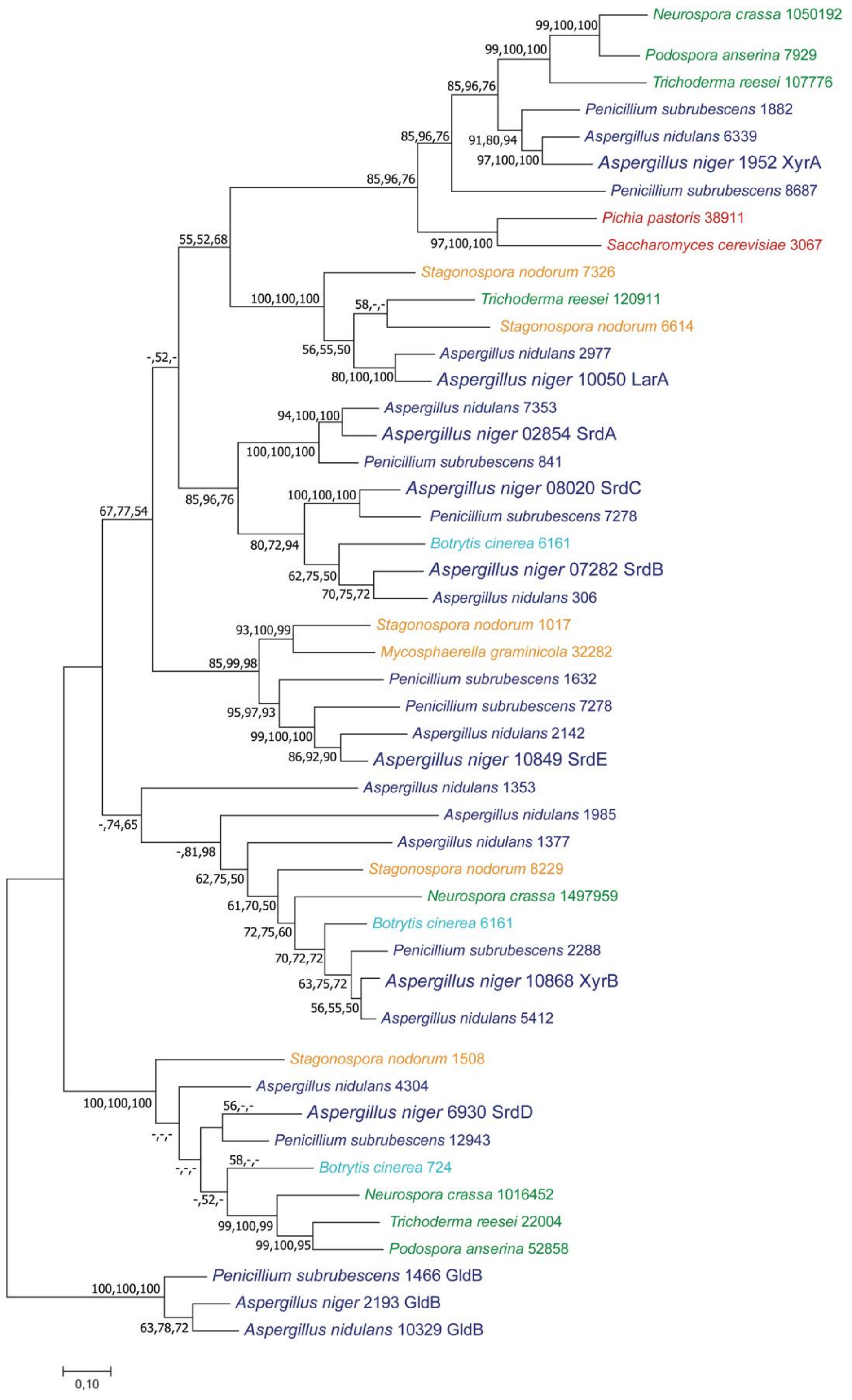

Supplemental Figure S1: Phylogenetic analysis of *A. niger* LarA, XyrA, XyrB, and their close homologues from 10 fungal species. Purple: Eurotiomycetes (*Aspergillus niger*, *Aspergillus nidulans* and *Penicillium subrubescens*), Green: Sordariomycetes (*Trichoderma reesei*, *Neurospora crassa, Podospora anserina*), Red: Saccharomycotina (*Saccharomyces cerevisiae* and *Pichia pastoris*), Orange: Dothideomycetes (*Mycosphaerella graminicola* and *Stagonospora nodorum*), and Turquoise: Leotiomycetes (*Botrytis cinerea*). The figure is a representative of a Maximum Likelihood (ML) tree (500 bootstraps) of a MAFFT alignment of the amino acid sequences. The first number on the node represent the ML bootstrap values. If the node also had a >50% support in a Neighboring Joining or Minimal Evolution tree, these values are represented by the second and third number, respectively. The numbers behind the species represent the protein ID in Mycocosm (https://mycocosm.jgi.doe.gov/mycocosm/home).

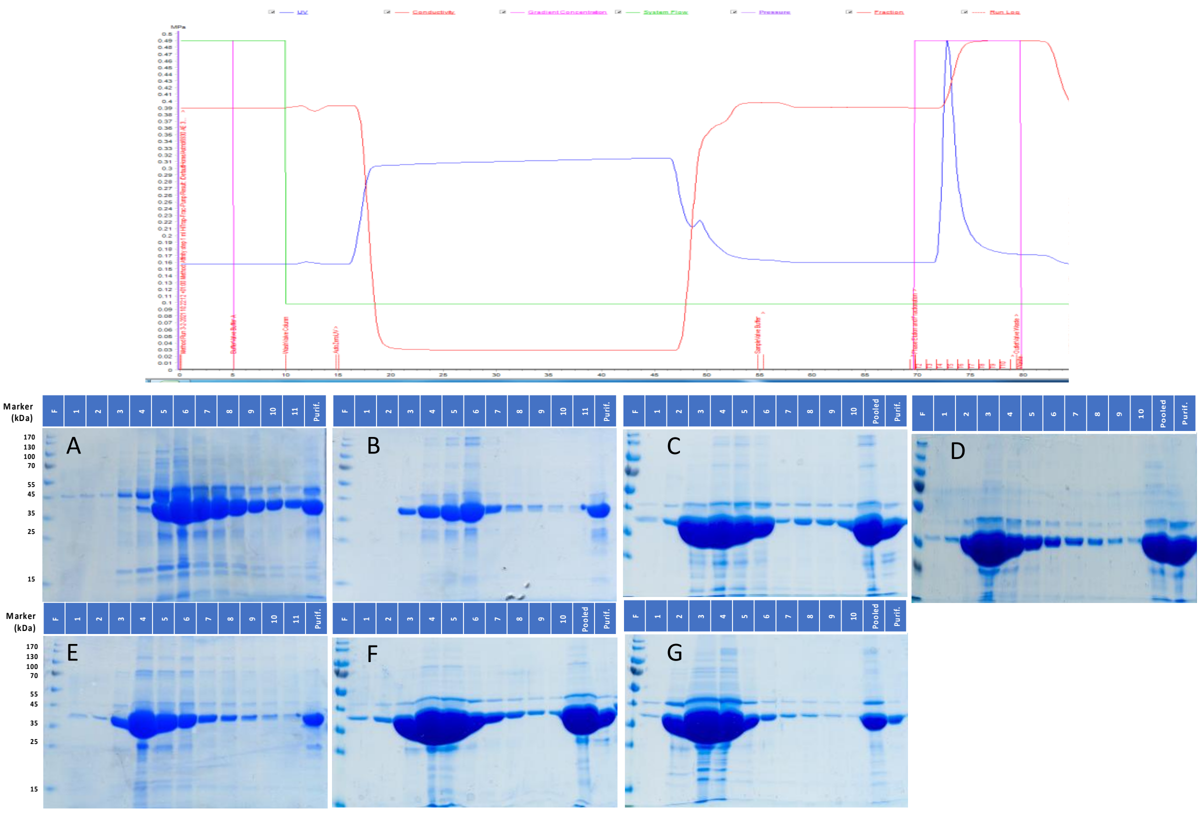

Supplemental Figure S2: Purification and verification of produced Srd's, LarA and XyrB proteins. Top, FPLC chromatogram with fractions/ absorbance peak at 280 nm. Bottom, fractions of purified recombinant proteins verified by SDS-PAGE. A: SrdA (2854), B: SrdB (7451), C: SrdC (8020), D: SrdD (6930), E: SrdE (10849), F: LarA (10050), G: XyrB (10868).

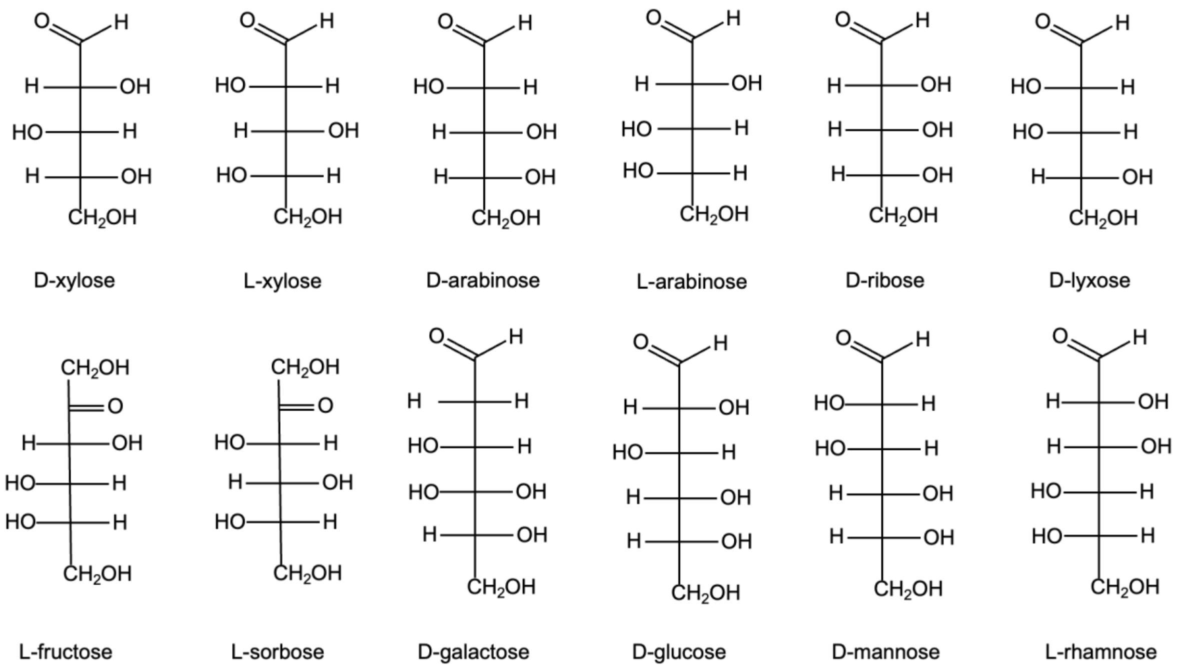

Supplemental Figure S3: Chemical structures (Fischer Projection) of monosaccharides used for biochemical characterization of putative sugar reductases.

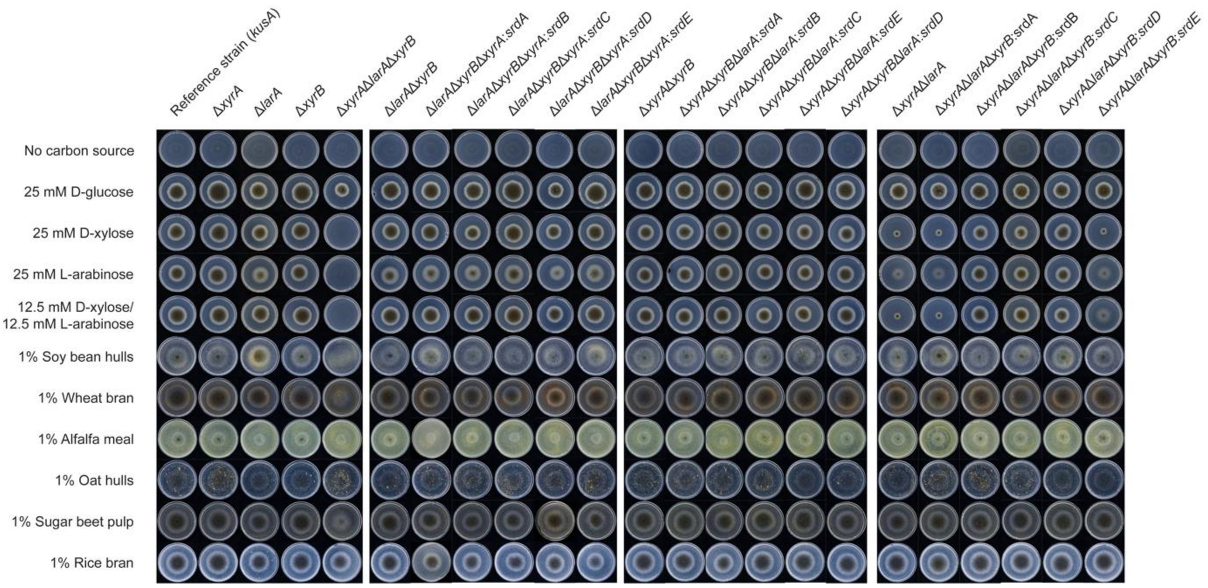

Supplemental Figure S4: Growth profile of the *A. niger* reference strain, and deletion and gene swaps strains of sugar reductases on monosaccharides (5 days growth) and crude substrates (4 days growth).

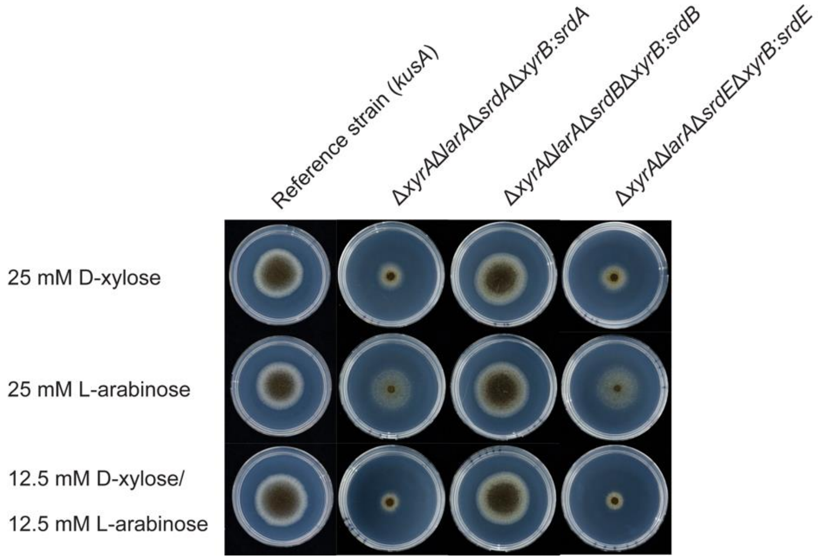

Supplemental Figure S5: Growth profile of the *A. niger* reference strain, and native copy deletion of gene swaps strains in the parental strain Δ*xyrA*Δ*larA* for Δ*xyrB:srdA*, Δ*xyrB:srdB* and Δ*xyrB:srdE*.

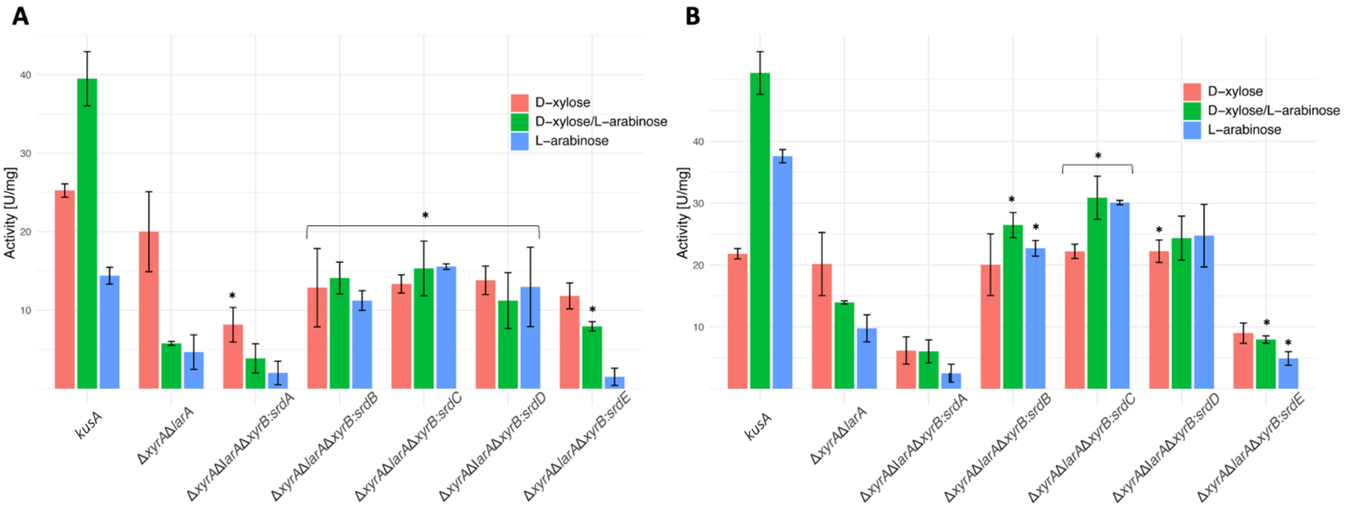

Supplementary Figure S6. Intracellular pentose reductase activity on D-xylose (A) and L-arabinose (B) of the *A. niger* reference strain Δ*ku70*, double deletion strain Δ*xyrA*Δ*larA* with gene swap mutants grown on D-xylose, L-arabinose, and D-xylose/L-arabinose (X/A). Statistical reference for putative gene swaps into the corresponding double deletion strain Δ*xyrA*Δ*larA* is indicated by an asterix, $p<0.05$.
